# Research on the Thermal Aging Characteristics of Cured Epoxy Resin Insulating Materials for DC Bushings

**DOI:** 10.3390/polym17081064

**Published:** 2025-04-15

**Authors:** Daijun Liu, Xiaobang Tong, Libao Liu, Tao Chen, Jiarong Tang, Wenkai Tang, Liming Wang, Bin Cao, Zimin Luo

**Affiliations:** 1 China Yangtze Power Co., Ltd., Beijing 100032, China; 2 Nanjing Electric High Voltage Bushing Co., Ltd., Nanjing 210046, China; 3 Shenzhen International Graduate School, Tsinghua University, Shenzhen 518055, China

**Keywords:** epoxy resin, thermal aging, insulating performance, heat resistance performance, mechanical performance

## Abstract

High-temperature-resistant epoxy composites play a crucial role in enhancing the operational reliability and service life of devices such as DC bushings, which is of great significance for the long-term stable operation of ultra-high voltage and flexible power transmission and distribution systems. In this study, the epoxy composite was prepared, and long-term thermal aging tests were conducted at 250 °C and 270 °C. The changes in physical properties, electrical characteristics, and bending strength of epoxy composite were systematically investigated, and the thermal aging mechanism of these materials was elucidated. The experimental results revealed that with the progression of thermal aging, the epoxy composites exhibited volume shrinkage due to the breaking of chemical bonds. After 10 thermal aging cycles at 270 °C, the mass loss rate of the epoxy composite reached 20.52%. At 250 °C, the breakdown strength decreased by 9.9% compared to the unaged state. After aging at 250 °C and 270 °C, the volume resistivity decreased by a maximum of 53.75% and 76.94%, respectively, while the dielectric constant decreased by a maximum of 50.34% and 41.94%, respectively. After 10 aging cycles at 250 °C and 270 °C, the bending strength of the cured epoxy composite decreased by 39.79% and 53.91%, respectively. These findings provide valuable insights into the thermal aging characteristics of epoxy composites used in DC bushings and other electrical devices, offering a scientific basis for material selection and reliability assessment in high-voltage insulation applications.

## 1. Introduction

Epoxy resins and their composites exhibit excellent insulation, high-temperature resistance, and fire-retardant performance [1,2,3] and are widely used in electrical equipment such as direct-current bushings. However, with the development trend of high integration and high power density of electrical equipment, the heat generated during operation accumulates inside the equipment, increasing the operating ambient temperature [4,5]. The poor thermal conductivity of epoxy resins in DC bushings makes it difficult to dissipate the accumulated heat inside the equipment, further increasing the operating ambient temperature of the bushings [6,7]. Long-term operation in high-temperature and electric field environments subjects epoxy resin to severe thermal performance tests. Research on the thermal aging mechanism of epoxy resins is crucial for enhancing their thermal performance and ensuring the reliable operation, lifetime assessment, and prediction of electrical equipment such as DC bushings [8,9,10,11].

The thermal aging of epoxy composites is affected by aging temperature, time, and oxygen content [12,13]. Reference [14] examined the accelerated thermal aging of pure epoxy and 3D carbon fiber/epoxy composites at different temperatures. It was found that high-temperature aging causes oxidative degradation of epoxy resin, forming an oxide layer on the surface. This layer hinders oxygen diffusion, protecting the internal resin from further oxidation. During thermal aging, the post-curing effect from heating and the thermal degradation of epoxy resin continuously compete, affecting the properties of epoxy composites. Reference [15] studied the accelerated thermal aging of epoxy resin at 100 °C, 130 °C, and 160 °C, characterizing the changes in its properties during aging. The study found that after high-temperature aging, the molecular chains of epoxy resin are broken down by high temperatures. The resin is oxidized, leading to mass loss and reduced insulation capability. During the aging process, the concentration of internal dipoles in epoxy resin increases, and polarization loss also rises, resulting in increased permittivity and dielectric loss. However, prior studies have focused on aging temperatures below the glass transition temperature (Tg) of epoxy resin. As shown in Table 1, even with extended aging durations, low aging temperatures fail to expose the end-of-life characteristics of epoxy resin. Thus, ultra-high-temperature accelerated aging tests above the Tg are urgently needed to investigate the complete aging failure process of epoxy resin.

Currently, domestic and international scholars focus mainly on the damage initiation and degradation characterization of epoxy resin thermal aging when studying the thermal aging mechanism of epoxy composites. Performance changes during thermal aging, such as mechanical [16,17], physical [18], thermal conductivity [19], and microcrack development [20], have been widely studied. However, previous research has focused on aging temperatures below the Tg of epoxy resin. The degradation mechanisms of epoxy resin composites during ultra-high-temperature thermal aging above their Tg remain to be explored.

This study prepared a thermally resistant epoxy composite and conducted ultra-high-temperature aging tests above its Tg (250 °C and 270 °C) in accordance with GB/T 11026.1-2016 [21]. Systematic tests were carried out to investigate the variation patterns of the insulation, thermal, and mechanical performance with thermal aging time. Through microstructural analysis, we revealed the performance degradation mechanism of the epoxy composite in ultra-high-temperature environments. This study hypothesizes that under ultra-high-temperature conditions, the aging rate of epoxy resin performance is significantly accelerated, and its aging process is still predominantly governed by molecular chain scission. This study provides an effective reference for the ultra-high-temperature aging characteristics and mechanisms of epoxy resin, filling the gap in existing research. Moreover, it offers vital theoretical foundations and data support for the condition assessment and lifespan prediction of electrical equipment, such as high-voltage DC bushings operating in high-temperature environments.

## 2. Materials and Methods

The raw materials used for the preparation of cured epoxy resin insulating materials are shown in Table 2.

In this paper, phthalic anhydride, hexahydrophthalic anhydride, and tetrahydrophthalic anhydride were used as blended curing agents to prepare epoxy resin samples modified by different amounts of 2-toluene glycidyl ether. The formulations are shown in Table 3. Based on the calculation formula of the amount of anhydride and epoxy resin, the mass required for the reaction of the blended anhydride curing agent with 100 g of epoxy resin was calculated. The calculation formula is shown in Equation (1) [22,23,24,25].

(1) $$W_{h}=M_{h}\times E_{v}\times k,$$

Here, *W_h_* represents the amount of curing agent used; *M_h_* is the relative molecular mass of the curing agent; *E_v_* is the epoxy value of the epoxy resin; and *k* is the proportionality coefficient. When a curing accelerator is used in the epoxy/anhydride curing system, *k* = 1; when no curing accelerator is used in the epoxy/anhydride curing system, *k* = 0.85. In this paper, *k* is taken as 1. The raw material ratios of the four anhydride curing agents with the epoxy resin curing system are shown in Table 3.

This paper employs a gravity casting method that is the same as that used in commercial bushings. The preparation process of epoxy resin is as follows:

1. Pre-treatment of casting mold: Place the epoxy resin casting mold to be used into a vacuum drying oven for preheating for 2 h at a temperature of 80 °C. After preheating, remove the mold and apply an appropriate amount of release agent on the surface in contact with the epoxy resin, then place it back into the vacuum drying oven for heat preservation and standby.
2. Pre-treatment of raw materials: Weigh the epoxy resin, curing agent, and accelerator according to the formula table, and place them in a vacuum drying oven at a temperature of 100 °C for drying and preheating for 0.5 h to obtain the dried liquid epoxy resin, curing agent, and accelerator for later use.
3. Preparation of epoxy resin casting material: Place the dried and preheated epoxy resin and curing agent into a reaction vessel heated and insulated by a heat preservation sleeve for vacuum stirring. The stirring is carried out using a high-speed disperser at a speed of 350 r/min, and a vacuum pump is used for continuous vacuum treatment during the stirring process, with a stirring time of 30 min. After stirring, add the weighed accelerator to the reaction vessel and continue stirring for 3 min under the same conditions to obtain the epoxy resin casting material.
4. Casting: Use the gravity casting method to pour the epoxy resin casting material into the prepared mold, and place the mold into a hot air drying oven for vacuum degassing treatment to remove the bubbles remaining in the resin and at the interface between the tree branch and the mold, with a vacuum treatment time of 5 min.
5. Curing: After vacuum degassing treatment, restore the hot air drying oven to atmospheric pressure for 16 h. After curing is completed, demold to obtain the epoxy resin sample.

**Figure 1:**
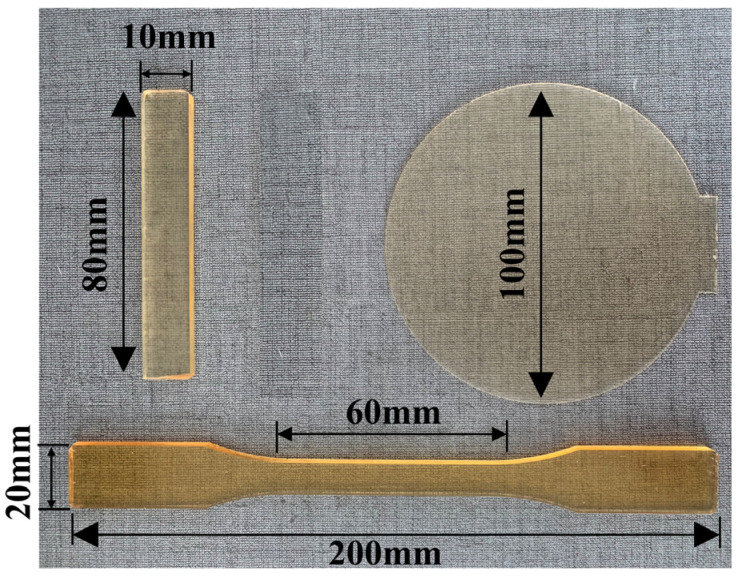
Dimensions of the epoxy resin specimen.

Samples used for breakdown electric field strength, volume resistivity, dielectric constant, and dielectric loss measurements have a thickness of 1 mm and a diameter of 100 mm. Samples for flexural strength testing are 80 mm in length, 10 mm in width, and 4 mm in thickness. Samples for tensile strength testing are 200 mm in length, 20 mm in width, and 4 mm in thickness, with a parallel middle section of 60 mm in length. The dimensions of the cured epoxy resin are illustrated in Figure 1.

## 3. Test Methods

### 3.1. Thermal Aging Test

According to the test standards specified in the national standard GB/T 11026.1-2016, an ultra-high-temperature test chamber was used to conduct thermal aging experiments on the prepared epoxy resin curing samples. The ultra-high-temperature test chamber was purchased from Dongguan Ke Zheng Instrument Co., Ltd. of Dongguan, China. The temperature gradients were set at 250 °C and 270 °C. The samples were aged for 48 h at 230 °C as a pre-aging step, and the mass of the samples was recorded at this point. Under the condition of 250 °C, the performance of the cured epoxy resin was tested every 3 days, with each test constituting one round. Under the condition of 270 °C, the performance of the cured epoxy resin was tested every 1 day, with each test constituting one round. Ten rounds of testing were conducted for each temperature.

The selection of ultra-high-temperature aging for epoxy resin is primarily aimed at accelerating the aging process, thereby shortening the experimental duration. Moreover, conducting aging tests at this temperature can offer dependable experimental data for predicting the service life of epoxy resin.

After the samples have reached the corresponding aging time, the samples are removed and cooled to room temperature, then weighed using an analytical balance from Mettler-Toledo International Inc. in Columbus, OH, USA. The mass measured each time is compared with the initial mass before aging, and the mass loss of the samples is calculated according to Equation (2).

(2) $$\eta_{mi}=\frac{m_{0}-m_{i}}{m_{0}}.$$

Here, $m_{0}$ is the initial mass of the sample; $m_{i}$ is the mass of the sample exposed to the environment at temperature $T_{i}$ for time $t_{i}$; $\eta_{mi}$ is the mass loss of the sample exposed to the environment at temperature $T_{i}$ for time $t_{i}$; and i is the sample number.

A Fourier Transform Infrared Spectrometer (FT-IR) from Thermo Nicolet was used to perform infrared tests on cured epoxy resin that underwent aging tests at different times and temperatures. The tests used KBr pellets with a wavelength range of 4000–1000 cm^−1^.

The experiments employed a method of calculating the mean value from five effective replicate samples to obtain the final data.

### 3.2. Insulating Performance Test

According to the test method of the national standard GB/T 1408.1-2016 [26], an AC breakdown strength test platform for epoxy resin insulating materials was set up. The breakdown voltage test platform consists of a power frequency power supply, a voltage regulator, a capacitive voltage divider, a water resistor, spherical electrodes, and an oil bath container. The electrodes used are spherical-spherical electrodes with a diameter of 20 mm. During the test, the epoxy resin sample is placed in insulating oil, and the test AC voltage rises uniformly at 2 kV/s until the epoxy resin sample is broken down, and the AC breakdown strength at this time is recorded.

According to the test method of the national standard GB/T 1410-2006 [27], the volume resistivity of the epoxy resin composite insulating material sample was tested. The resistivity test used a ZC46A high-resistance meter, and the test sample was a circular disk with a diameter of 100 mm and a thickness of 1 mm. The ZC46A high-resistance meter was from Shanghai Cany Precision Instrument Co., Ltd., Shanghai, China. The measurement environment had a humidity of 50% RH and a temperature of 25 °C. The test voltage was 1 kV, and the reading after applying the voltage for 60 s was recorded to calculate the volume resistivity of the sample.

According to the test method of the national standard GB/T 1409-2006 [28], the dielectric constant and dielectric loss of the cured epoxy resin insulating material were tested. The test used a QS-87 high-voltage bridge, and the test sample was a circular disk with a diameter of 100 mm and a thickness of 1 mm. The QS-87 high-voltage bridge was from Shanghai Yanggao Electrical Appliance Co., Ltd., Shanghai, China. The test voltage was 1 kV, and the frequency was 50 Hz. The dielectric constant and dielectric loss of the cured epoxy resin insulating material were obtained from the test.

The tests for breakdown strength, dielectric constant and dielectric loss factor used five effective replicate samples, while volume resistivity used 3–4 effective replicate samples, and the final results were obtained by calculating the mean values.

### 3.3. Heat Resistance Test

According to the test method of the international standard ISO 11357-2-2014 [29], the glass transition temperature of the epoxy resin insulating material was tested. The test was conducted using a TAQ2000 differential scanning calorimeter with a heating rate of 10 °C/min, heating from room temperature to 250 °C to obtain the glass transition temperature of the cured epoxy resin. The TAQ2000 differential scanning calorimeter was from TA Instruments Co., Ltd., New Castle, DE, USA.

According to the test method of the international standard ISO 11358-2-2014 [30], a thermogravimetric analysis test was conducted on the epoxy resin insulating material. The test was performed using a TAQ500 thermogravimetric analyzer in an air atmosphere with a temperature range of 40~700 °C and a heating rate of 10 °C/min to obtain the thermogravimetric analysis curve of the cured epoxy resin. The TAQ500 thermogravimetric analyzer was from TA Instruments Co., Ltd., New Castle, DE, USA.

The experiments employed a method of calculating the mean value from five effective replicate samples to obtain the final data.

### 3.4. Mechanical Performance Test

According to the test method of the national standard GB/T 2567-2008 [31], the bending strength and tensile strength of the epoxy resin insulating material were tested. The test was conducted using a universal mechanical testing machine with a loading speed of 2 mm/min to obtain the bending strength and tensile strength of the cured epoxy resin. The universal mechanical testing machine was from Jinan Wenteng Test Instrument Co., Ltd., Jinan, China.

The experiments used a method of calculating the mean value from five effective replicate samples to obtain the final data.

## 4. Results and Discussion

### 4.1. Physical Performance of Cured Epoxy Resin

**Figure 2:**
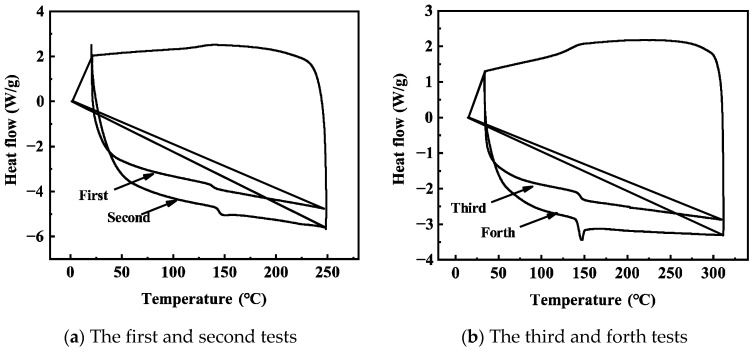
DSC of cured epoxy resin before aging.

In order to determine whether ultra-high-temperature aging leads to the post-curing of epoxy resin, we conducted DSC tests on unaged epoxy resin. The same sample was tested four times, and the results are shown in Figure 2. The T_g_ measured in the four tests was 147.23 °C, 149.02 °C, 149.31 °C, and 149.28 °C, respectively. The results indicate that the maximum increase in T_g_ in the last three tests compared to the first test was only 2.08 °C, leading us to conclude that the epoxy resin was almost completely cured. Under the aging conditions of 250 °C/3 days or 270 °C/1 day in the first round of aging, any uncured parts would rapidly cure and initiate the aging of the epoxy resin. Therefore, the post-curing process in the aging test is very brief, and the main process is the aging of the epoxy resin.

**Figure 3:**
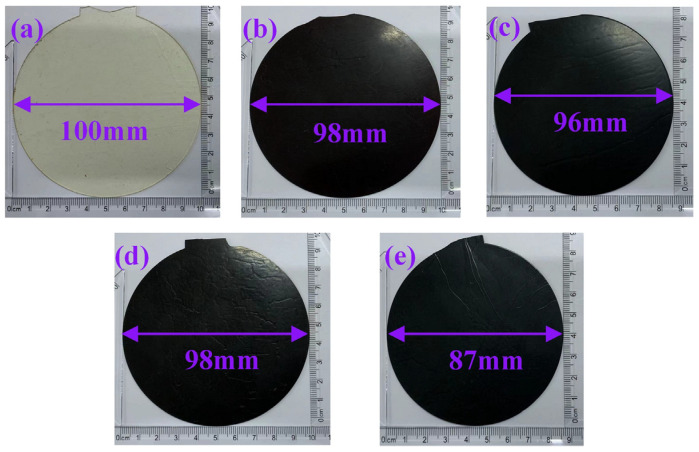
Structure change in cured epoxy resin after thermal aging: (**a**) unaged, (**b**) 250 °C aging for one cycle, (**c**) 250 °C aging for ten cycles, (**d**) 270 °C aging for one cycle, (**e**) 270 °C aging for ten cycles.

Thermal aging tests were conducted on cured epoxy resin at different temperatures, and the morphology of the cured epoxy resin before and after aging is shown in Figure 3. The test results show that the unaged cured epoxy resin is a light yellow, transparent, thin sheet with a smooth surface. After the first round of aging, the cured epoxy resin turns into a black, opaque, thin sheet, and the composite aged at 270 °C shows shrinkage wrinkles on the surface. After the tenth round of aging, the color of the cured epoxy resin darkens, and the surface shrinkage marks become more pronounced. The color change in the cured epoxy resin indicates that chemical reactions have occurred on the surface of the epoxy resin due to thermal oxidation, including post-curing and thermal decomposition. When the thermal aging temperature is above the glass transition temperature of the cured epoxy resin, the thermal decomposition process is predominant.

**Figure 4:**
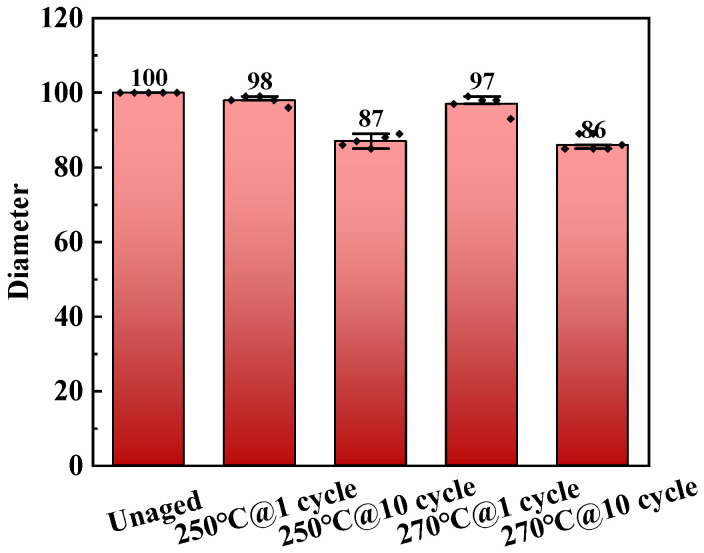
Diameter of epoxy resin before and after thermal aging.

We calculated the changes in the diameter of the samples before and after aging. We measured the diameters of five samples and used their average value as the final contracted diameter. The measurement data of the diameters before and after aging are shown in Figure 4. The test results show that the diameters of the unaged samples have no variability, which is due to the fact that we used the same mold to prepare all the samples. The diameters of the aged samples have a small degree of dispersion, which indicates the reliability of using diameter measurements to assess the structural contraction of the epoxy resin.

The diameter change rate of the cured epoxy resin before and after thermal aging was recorded, and the calculation results are shown in Table 4. The results show that as the aging time increases, the diameter shrinkage rate of the cured epoxy resin continuously increases; as the aging temperature increases, the diameter shrinkage rate of the cured epoxy resin also increases. The increase in aging temperature and time leads to a greater degree of decomposition of the cured epoxy resin, resulting in an increased diameter shrinkage rate. When the aging temperature is 250 °C and 270 °C, respectively, and after ten rounds of aging, the diameter of the cured epoxy resin is reduced by 13.00% and 14.00%, respectively, which will seriously affect the reliable and safe operation of electrical equipment.

**Figure 5:**
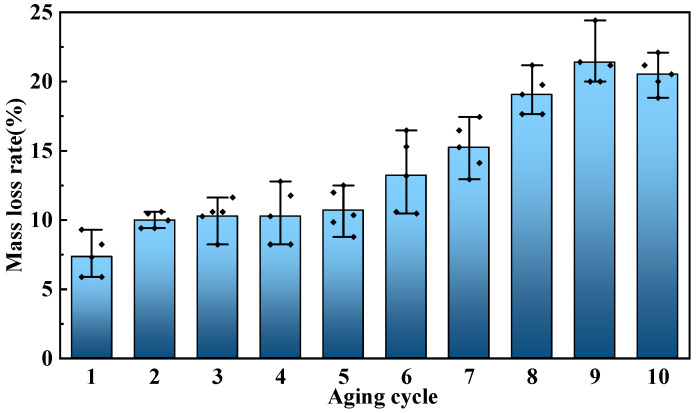
Mass loss rate of cured epoxy resin at 270 °C.

The variation in the mass of the cured epoxy resin with aging temperature and increasing aging time was recorded, and Figure 5 shows the mass loss rate of the cured epoxy resin with aging time at 270 °C. The test results show that as aging progresses, the mass loss rate of the cured epoxy resin also continuously increases, reaching 20.52% by the tenth round of aging. In the early stages of aging, the water and residual small molecules inside the cured epoxy resin continuously evaporate under the influence of high temperature. As aging progresses, the cured epoxy resin undergoes oxidative decomposition, leading to a continuous decrease in mass and a rising mass loss rate.

**Figure 6:**
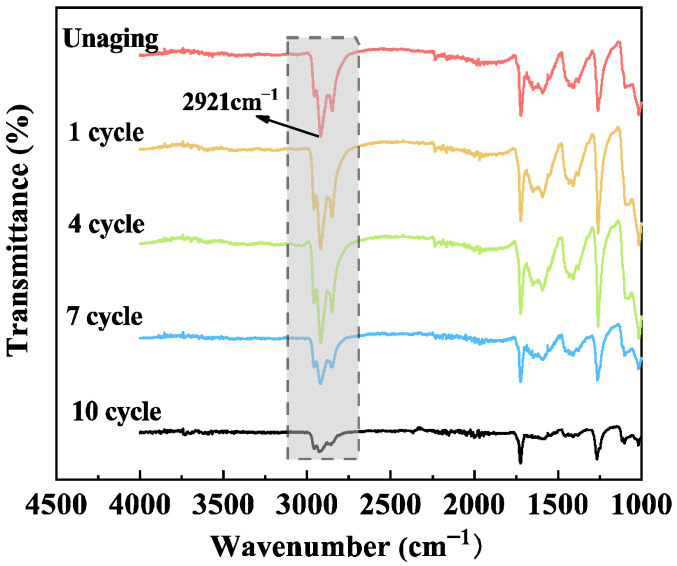
Infrared test of cured epoxy resin after thermal aging at 270 °C.

Figure 6 shows the infrared detection results of the surface of cured epoxy resin after different aging times at 270 °C. For ease of comparison, the spectral lines are shifted vertically to overlap their baselines. The detection results show that the transmittance of the infrared spectral absorption bands near the wavenumbers of 2921 cm^−1^ and 2870 cm^−1^ continuously decreases with the aging time. The comparison between the unaged sample and the sample after 10 rounds of aging is more evident. The change in transmittance of different samples in infrared testing indicates that the C-H bonds on the methyl or methylene groups between two phenyl rings are continuously being destroyed and broken by high temperature. C-H bonds have lower bond energy. They are destroyed during ultra-high-temperature aging. This causes the surface of cured epoxy resin to oxidize. As a result, the various properties of cured epoxy resin are affected to varying degrees.

### 4.2. Insulating Performance of Cured Epoxy Resin

**Figure 7:**
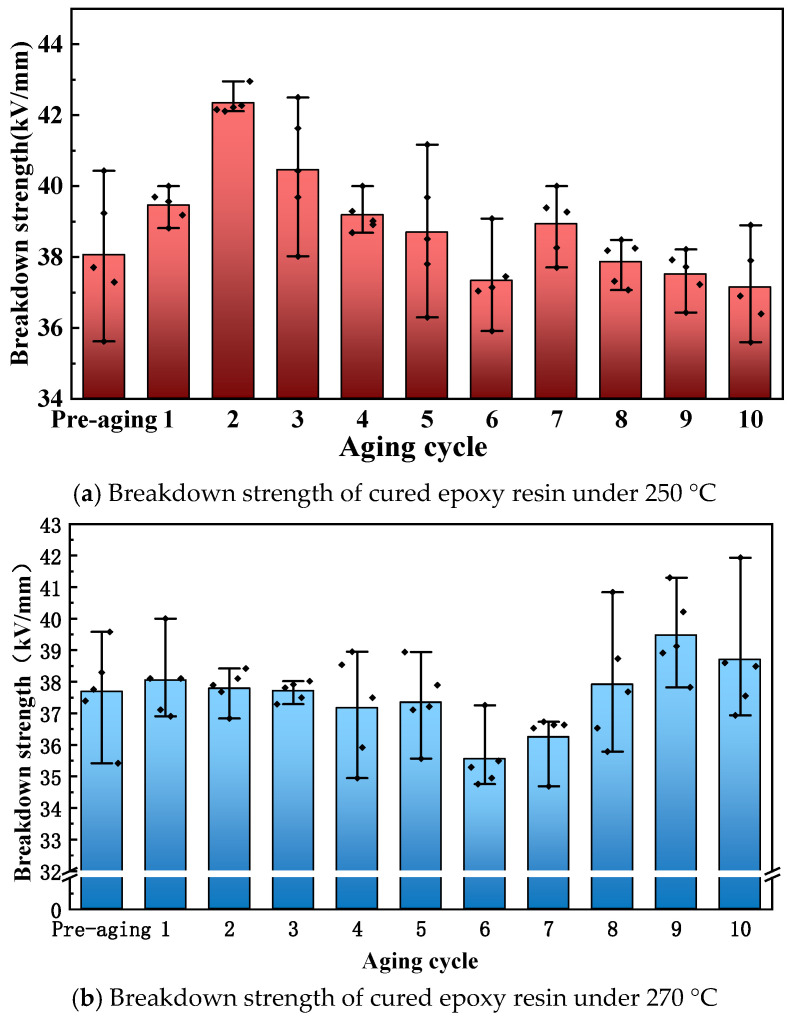
Breakdown strength of cured epoxy resin after thermal aging.

Figure 7 shows the breakdown strength test results of cured epoxy resin after aging at different temperatures and times. When the thermal aging temperature is 250 °C, the breakdown strength of the cured epoxy resin first increases and then continuously decreases with the aging process. By the 10th round of aging, the breakdown strength of the cured epoxy resin has decreased by 9.9% compared to the unaged state (the breakdown strength of the initial cured epoxy resin is 41.22 kV/mm). In the early stage of thermal aging, the breakdown strength of cured epoxy resin increases. This is attributed to ultra-high-temperature thermal degradation, which creates a charge barrier on the material’s surface. This barrier hinders charge migration within the cured epoxy resin, thereby enhancing its breakdown strength. However, as aging continues, the material suffers severe high-temperature damage. Local structural fractures and gap defects emerge, significantly reducing the breakdown strength of the cured epoxy resin.

When the thermal aging temperature is 270 °C, the change in the breakdown strength of the cured epoxy resin is not significant, with a decrease only after the 6th and 7th rounds of aging. Compared to the unaged state, the breakdown strength of the cured epoxy resin decreased by 8.42% after pre-aging and by 6.11% after 10 rounds of aging, indicating a small change in breakdown strength throughout the ten rounds of aging. After pre-aging at a higher temperature, an oxide layer forms on the surface of the cured epoxy resin, preventing air from entering the interior and reducing the ultra-high-temperature oxidation of the internal cured epoxy resin, thus preventing a significant decrease in breakdown strength with aging. In the later stages of aging, the shrinkage of the dimensions due to thermal aging causes the thickness of the cured epoxy resin to decrease and the structure to become more compact, which to some extent increases the breakdown strength of the cured epoxy resin.

**Figure 8:**
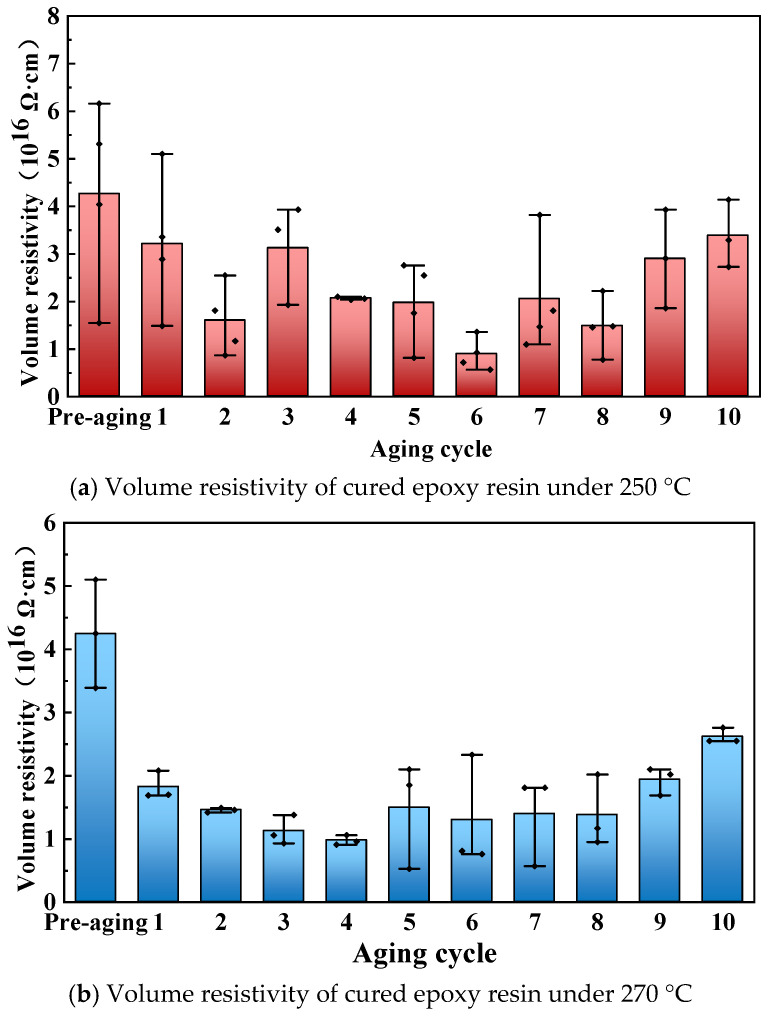
Volume resistivity of cured epoxy resin at different aging times.

The volume resistivity of cured epoxy resin was tested, and the variation pattern of volume resistivity after different aging times at different aging temperatures is shown in Figure 8, where the aging cycle of 0 refers to the test result after pre-aging. The test results show that the volume resistivity of cured epoxy resin generally shows a trend of first decreasing and then increasing with the progress of aging, but the change is limited, and it still remains in the same order of magnitude. Thermal aging involves chemical-bond recombination and breaking. The recombination causes post-curing of cured epoxy resin, while the breaking leads to its thermal decomposition.

When the aging temperature is 250 °C, the volume resistivity of cured epoxy resin drops significantly after the first round of aging and then shows fluctuating changes with the progress of aging cycles. When the aging temperature is 270 °C, the decrease in volume resistivity after the first round of aging is greater than that at the 250 °C aging condition, which is due to the increased aging temperature and the increased degree of aging of cured epoxy resin. As aging continues, the volume resistivity of cured epoxy composite remains relatively stable. This is because the oxide layer formed during the first aging cycle protects the interior from oxidation, preventing significant volume resistivity changes.

**Figure 9:**
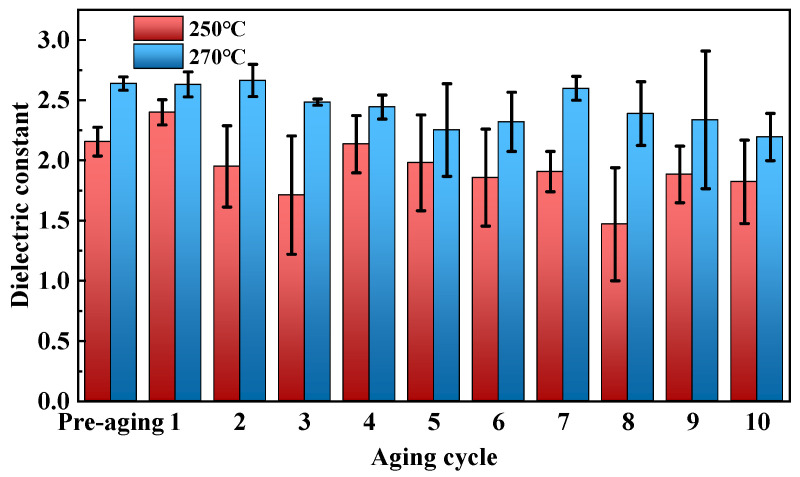
Dielectric constant of cured epoxy resin after thermal aging.

We tested the dielectric constant and dielectric loss factor of epoxy composites under power frequency conditions. Figure 9 shows the dielectric performance of cured epoxy resin after thermal aging tests at various temperatures and durations. Results indicate that at 250 °C and 270 °C, the dielectric constant and dielectric loss factor of cured epoxy resins initially decrease, then increase, and finally decrease again as aging time extends.

In the early stage of thermal aging, the higher aging temperature causes partial decomposition of polar groups in the cured epoxy resin, leading to a decrease in its dielectric constant. In the middle stage of thermal aging, ions in the cured epoxy resin are thermally activated by the temperature and accelerate their transport, resulting in an upward trend in the dielectric constant of the cured epoxy resin during the middle stage of aging. In the late stage of thermal aging, the cured epoxy resin continues to degrade thermally. The diameter contraction of the cured epoxy resin reduces the migratable space for charge carriers such as ions, leading to a decrease in the dielectric constant of the cured epoxy resin.

**Figure 10:**
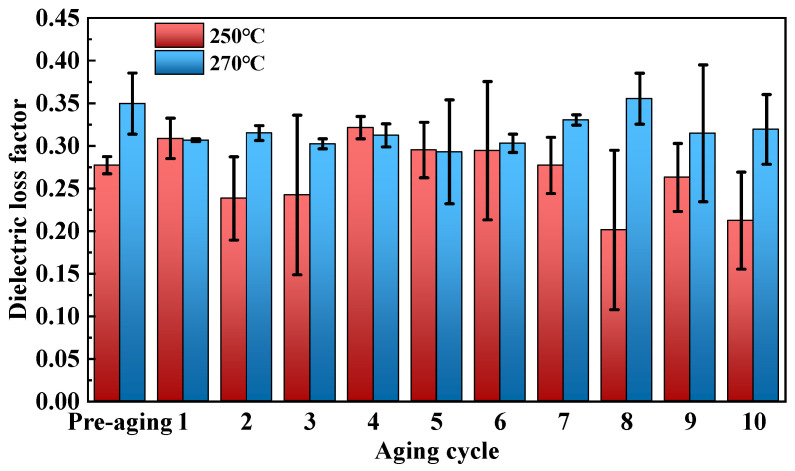
Dielectric loss factor of cured epoxy resin after thermal aging.

The dielectric loss factor test results are shown in Figure 10, and the variation trend of the dielectric loss factor of cured epoxy resin at different aging temperatures is almost consistent with their dielectric constant. The dielectric loss of cured epoxy resin under power frequency mainly consists of conduction loss and polarization loss. In the early stage of aging, the thermal decomposition of polar groups leads to a decrease in polarization loss caused by the rotation of dipoles, thereby reducing the dielectric loss factor of cured epoxy resin. As aging progresses, ion transport becomes more intense due to temperature excitation, thereby generating Joule heat and increasing the dielectric loss factor of cured epoxy resin. In the later stage of aging, the transport of ions and other charge carriers is hindered due to the contraction of migratable space, and the thermal loss generated by charge carrier transport decreases, leading to a downward trend in the dielectric loss factor of cured epoxy resin.

### 4.3. Thermal Performance of Cured Epoxy Resin

**Figure 11:**
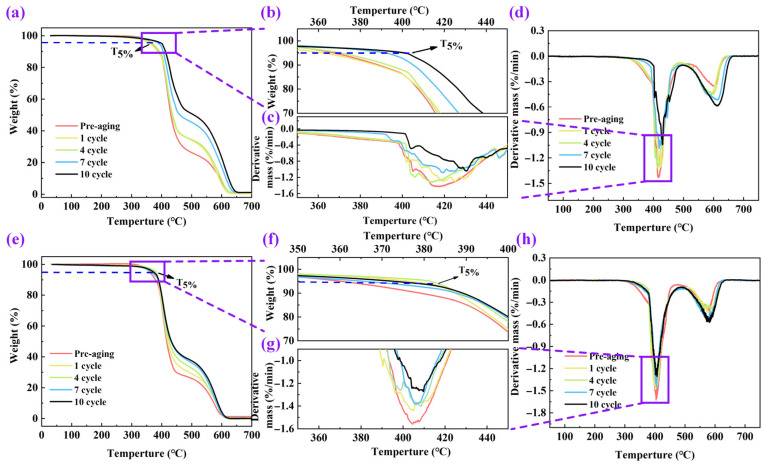
Thermogravimetric analysis of cured epoxy resin after thermal aging: (**a**,**b**) thermogravimetric curve and (**c**,**d**) thermogravimetric rate curve under 250 °C; (**e**,**f**) thermogravimetric curve and (**g**,**h**) thermogravimetric rate curve under 270 °C.

Figure 11 shows the mass variation curves of cured epoxy resin with temperature after aging under different temperatures and time conditions, analyzing the change patterns of the mass fraction and mass loss rate of the aged cured epoxy resin with the increase in temperature. As shown in Figure 11a,c, the thermogravimetric curves of cured epoxy resin at different aging times do not overlap significantly. However, the thermogravimetric rate curves of cured epoxy resin at different aging times shown in Figure 11b,d have a high degree of overlap.

The thermogravimetric analysis results of cured epoxy resin are shown in Table 5, where T_5%_ is the thermal decomposition temperature, referring to the temperature at which the mass of the cured epoxy resin decreases by 5%; T_10%_ is the temperature at which the mass of the cured epoxy resin decreases by 10%; T_MAX_ is the temperature at which the mass loss rate of the cured epoxy resin is the highest; and Rw is the remaining mass fraction of the cured epoxy resin after thermal treatment. The test results show that when the thermal aging temperature is 250 °C, with the increase in the thermal aging cycles, the T_5%_ of the cured epoxy resin first increases and then decreases, while its T_10%_ continuously increases and eventually tends to saturate. When the thermal aging temperature is 270 °C, with the increase in the thermal aging cycles, the T_5%_ and T_10%_ of the cured epoxy resin continuously increase, and compared to the aging condition at 250 °C, the T_5%_ and T_10%_ of the cured epoxy resin aged for the same number of cycles at 270 °C are larger. This is because after higher thermal treatment temperatures and longer thermal treatment times, the cured epoxy resin undergoes longer thermal decomposition, and its mass loss continues to increase, resulting in the need for higher temperatures to decompose the cured epoxy resin, thereby increasing the T_5%_ and T_10%_ of the cured epoxy resin. The test results of T_MAX_ show that under different aging temperatures, after different aging times, the temperature at which the mass loss rate of the cured epoxy resin is the highest is almost the same. The test results of Rw show that after thermal treatment, the remaining mass fraction of the cured epoxy resin continuously decreases with the extension of aging time. When the aging temperature is 270 °C, compared to the pre-aged cured epoxy resin, its Rw decreases by 46.5% after 10 rounds of aging, indicating that thermal aging has a significant effect on the mass loss of the cured epoxy resin.

### 4.4. Mechanical Performance of Cured Epoxy Resin

**Figure 12:**
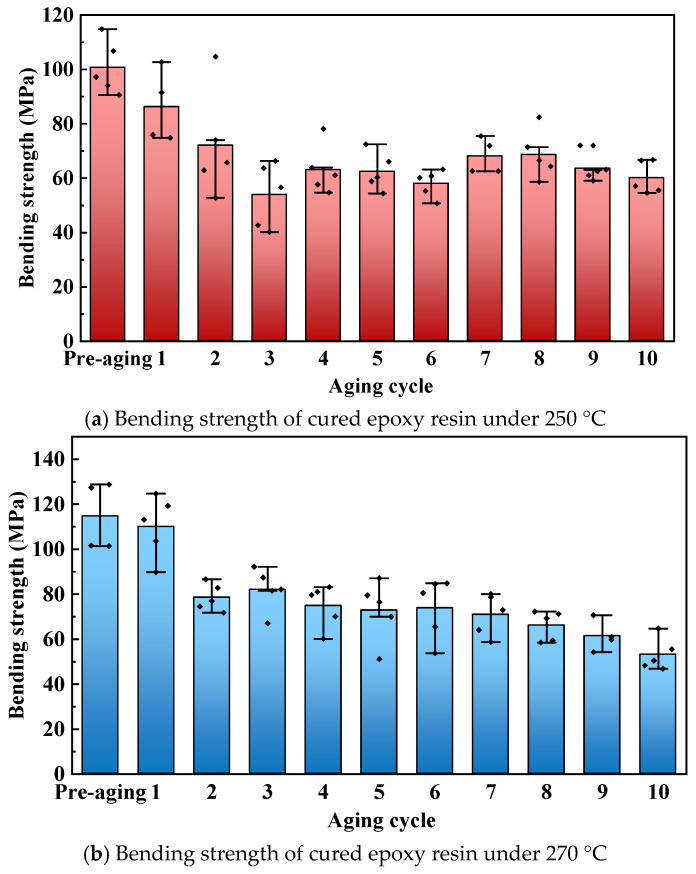
Bending strength of cured epoxy resin at different aging times.

Bending strength tests were conducted on cured epoxy resin after thermal aging at different temperatures and times, with the results shown in Figure 12, where an aging cycle of 0 refers to the cured epoxy resin after pre-aging. The test results show that the bending strength of cured epoxy resin continuously decreases with the extension of aging time. Compared to the cured epoxy resin after pre-aging, when the aging temperature is 250 °C and 270 °C, the bending strength of the cured epoxy resin decreases by 39.79% and 53.91% after 10 rounds of aging, respectively. The thermal degradation at high temperatures destroys the cross-linked structure of the cured epoxy resin, reducing its ability to resist strain, leading to a significant decrease in bending strength. The bending strength of cured epoxy resin decreases rapidly during the first two rounds of aging, and the rate of decrease in bending strength significantly slows down in subsequent aging processes. This is because the oxide layer formed on the surface of the cured epoxy resin in the early stages of aging hinders the aging process from progressing into the interior of the composite. In the later stages of aging, the degradation rate inside the cured epoxy resin significantly decreases, leading to a gradual slowdown in the rate of decrease in bending strength.

### 4.5. Statistical Analysis of Performance Degradation of Epoxy Composite After Thermal Aging

**Figure 13:**
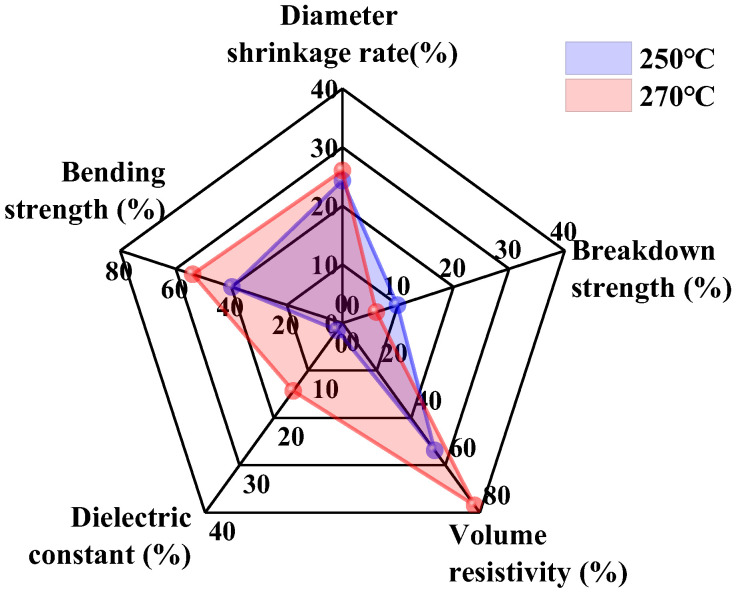
Statistical analysis of performance degradation of epoxy composite.

The performance degradation of epoxy composites after 10 rounds of ultra-temperature thermal aging at 250 °C and 270 °C was statistically analyzed, with results shown in Figure 13. This severe aging damages the molecular structure, significantly reducing the volume, volume resistivity, and bending strength of epoxy composite.

During the service of high-voltage DC bushings, the volume contraction of epoxy composites forms stress accumulation at the interface with the metal conductor. The decline in bending strength promotes interfacial defect development, a key cause of insulation breakdown. The substantial decrease in volume resistivity accelerates DC charge accumulation in epoxy composite, leading to local electric field distortion and faster insulation breakdown.

The high-current and high-temperature operating environment of high-voltage DC bushings imposes stringent requirements on epoxy resin material design. Based on the experimental results, when formulating epoxy composite recipes, their high thermal resistance, mechanical strength, and insulation properties should be emphasized. Firstly, design epoxy materials with higher initial volume resistivity, insulation strength, and bending strength to enhance the safety and service life of high-voltage DC bushings. Secondly, strictly control the operating temperature within the epoxy resin’s tolerance to prevent rapid aging from excessive thermal stress. Finally, enhance condition monitoring to promptly detect abnormal operating states and avoid equipment operation issues caused by early local defects.

## 5. Conclusions

This paper explores how the key electrical, thermal, and mechanical properties of cured epoxy resin change under ultra-high-temperature conditions. Accelerated thermal aging tests were conducted at varying temperatures and durations. The study aims to reveal the thermal aging mechanism and performance degradation of cured epoxy resin insulating materials. This provides a reliable and effective reference for the safe and stable operation of electrical equipment, such as DC bushings. The conclusions are as follows:

(1) The results of infrared spectroscopy show that the chemical bonds of cured epoxy resin materials are broken during the aging process, leading to surface degradation and consequently causing dimension shrinkage and mass loss of the cured epoxy resin. When the thermal aging temperature is 270 °C, after the tenth round of aging, compared with the unaged cured epoxy resin materials, the dimension shrinkage reaches 14.00%, and the mass loss reaches 20.52%.
(2) Thermal aging destroys the microscopic surface structure of the cured epoxy resin, reducing its electrical properties. After the tenth round of aging at a thermal aging temperature of 250 °C, the breakdown strength of the cured epoxy resin decreases by 9.9% compared to before aging, and the volume resistivity also drops significantly. Under different aging temperatures, the dielectric constant and dielectric loss of the cured epoxy resin fluctuate with the progress of aging, but the variation amplitude is relatively small.
(3) In the early stage of thermal aging, excessively high temperatures damage the crosslinked structure of the cured epoxy resin, causing a significant decrease in its flexural strength. However, as the oxidation layer forms on the surface of the cured epoxy resin, the penetration of oxygen is hindered, reducing the degree of internal aging of the cured epoxy resin, and thus the rate of decrease in its flexural strength tends to be gentle. Compared with the pre-aged cured epoxy resin, after ten rounds of aging at 250 °C and 270 °C, the flexural strength of the cured epoxy resin decreased by 39.79% and 53.91%, respectively.

## Figures and Tables

**Table 1:** Current status of thermal aging characteristics of epoxy resins at temperatures below their Tg.

Source	Tg (°C)	Aging Test Procedure	Changes in Key Performance
[4]	121	120 °C/2500 h	5.44% increase in flexural strength
[13]	114.9	80 °C/168 h	11.43% increase in AC breakdown electric field strength
[14]	110	90 °C/384 h	9% increase in compression
	110	120 °C/384 h	7% reduction in compression
[16]	140	160 °C/720 h	73.33% reduction in tensile strength

**Table 2:** Raw materials for cured epoxy resin insulating materials.

Name	Category	Specification	Origin
Bisphenol A Diglycidyl Ether	Epoxy Resin	Chemical Grade	Shanghai Kingchem Co., Ltd. (Shanghai, China)
Phthalic Anhydride	Curing Agent	Analytical Grade	Shanghai Macklin Biochemical Technology Co., Ltd. (Shanghai, China)
Hexahydrophthalic Anhydride	Curing Agent	Analytical Grade	Shanghai Macklin Biochemical Technology Co., Ltd. (Shanghai, China)
Tetrahydrophthalic Anhydride	Curing Agent	Analytical Grade	Shanghai Macklin Biochemical Technology Co., Ltd. (Shanghai, China)
2-Toluene Glycidyl Ether	Modifier	Chemical Grade	Shanghai Macklin Biochemical Technology Co., Ltd. (Shanghai, China)
*N*,*N*-Dimethylbenzylamine	Accelerator	Chemical Grade	Shanghai Macklin Biochemical Technology Co., Ltd. (Shanghai, China)

**Table 3:** Formula of epoxy resin insulating material modified by 2-toluene glycidyl ether.

Number	Epoxy Resin (g)	Blended Curing Agent (g)	2-Toluene Glycidyl Ether (g)	BDMA (g)
EP-HTP	100	81.8	0	0.6
EP-HTP-5	100	86.5	5	0.6
EP-HTP-10	100	91.1	10	0.6
EP-HTP-15	100	95.8	15	0.6

**Table 4:** Diameter change in cured epoxy resin after thermal aging.

Temperature (°C)	Diameter Before Aging (mm)	Diameter After 1 Cycle of Aging (mm)	Diameter Shrinkage Rate After 1 Cycle of Aging (%)	Diameter After 10 Cycles of Aging (mm)	Diameter Shrinkage Rate After 10 Cycles of Aging (%)
250	100	98	2.00	87	13.00
270	100	97	3.00	86	14.00

**Table 5:** Thermal performance of cured epoxy resin after thermal aging.

Aging Cycles	T_5%_ (°C)		T_10%_ (°C)		T_MAX_ (°C)		R_w_ (%)	
	250	270	250	270	250	270	250	270
Pre-aged	361.25	367.71	379.3	388.25	403.64	417.21	1.26	1.57
1	377.76	361.81	387.39	387.1	401.95	424.81	0.2	1.31
4	381.92	370.23	388.93	392.58	407.93	413.67	0.14	1.31
7	366.51	391.9	387.8	405.12	404.8	419.21	0.14	0.99
10	373.66	401.69	389.3	412.79	407.11	430.54	0.13	0.84

## Data Availability

The original contributions presented in this study are included in the article. Further inquiries can be directed to the corresponding authors.
